# Tuberculosis diagnosis using artificial intelligence: current trends and future prospects

**DOI:** 10.3389/fmed.2025.1569615

**Published:** 2026-01-07

**Authors:** Onesime Mbulayi, Saint-Jean Djungu, Loukia Aketi, Mohammed Amine Koulali, Hanae Azzaoui, Rim Koulali, Mohammed El Mzibri, Imane Chaoui, Yahya Tayalati

**Affiliations:** 1 Department of Mathematics, Statistics and Informatics, University of Kinshasa, Kinshasa, Democratic Republic of Congo; 2 Applied Informatics Research Center (CRIA), University of Kinshasa, Kinshasa, Democratic Republic of Congo; 3 Cardio-pulmonary and Infectious Diseases Unit, Department of Pediatrics, University of Kinshasa, Kinshasa, Democratic Republic of Congo; 4 Laboratoire de Modelisation et Calcul Scientifique (LMCS), ENSAO, Mohammed First University, Rabat, Morocco; 5 College of Computing, Mohammed VI Polytechnic University, Ben Guerir, Morocco; 6 Laboratoire Genie Electrique et Maintenance (LGEM), ESEF, Mohammed First University, Oujda, Morocco; 7 LIS Laboratory, Faculty of Sciences Ain Chock (FSAC), Hassan II University of Casablanca, Casablanca, Morocco; 8 Centre Nationale de l’Energie, des Sciences et des Technologies Nucleaires (CNESTEN), Rabat, Morocco; 9 Faculty of Sciences, University Mohammed V in Rabat, Rabat, Morocco; 10 School of Applied and Engineering Physics, Mohammed VI Polytechnic University, Ben Guerir, Morocco

**Keywords:** tuberculosis, mycobacterium, artificial intelligence, chest radiography, diagnosis

## Abstract

Tuberculosis, an infectious disease caused by *Mycobacterium tuberculosis*, poses a major global health challenge. Despite being largely controlled for several decades, tuberculosis has experienced a resurgence in recent years. China has the second highest incidence of tuberculosis globally, with a prevalence of 459 cases per 100,000 individuals aged 15 years old. Chest radiography and pathology are essential tools for its detection and diagnosis. However, the small size and low number of tubercle bacilli make detection and identification under a microscope challenging, often resulting in low detection rates and false diagnoses. Artificial intelligence (AI) has emerged as a promising tool to improve the accuracy and sensitivity of tuberculosis detection. This review provides a comprehensive overview of the literature on the use of machine learning-based models for the automatic detection of tuberculosis bacilli, emphasizing the advantages of integrating in tuberculosis diagnosis. Understanding the onset and progression of tuberculosis is crucial to developing effective strategies for its diagnosis, treatment, and prevention.

## 1 Introduction

Worldwide, tuberculosis, caused by *Mycobacterium tuberculosis*, remains a global public health challenge. Despite a significant decline in incidence due to effective control measures over the preceding decades, tuberculosis has experienced a resurgence on a global scale, particularly heightened by socio-economic factors and the human immunodeficiency virus (HIV) epidemic (1). Before the onset of the COVID-19 pandemic, tuberculosis was the leading cause of mortality worldwide, with nearly 1.4 million deaths in 2019 alone (1).

Several countries have significantly contributed to the global burden of tuberculosis incidence, as evidenced by comprehensive national surveys. These surveys reported notable prevalence rates of active tuberculosis in populations aged ≥ 5 years (2). Given these figures, the effective management and treatment of tuberculosis have become an indispensable responsibility for healthcare providers. Notably, a substantial proportion of tuberculosis patients are asymptomatic, which underscores the critical role of chest radiography in disease detection and diagnosis (3, 4). Furthermore, pathological examination remains the cornerstone of clinical tuberculosis diagnosis, necessitating the identification of specifically stained tubercle bacilli and associated morphological tissue changes under microscopic analysis (5).

The detection and identification of these bacilli are challenged by their minute size, less than 1 μm in diameter, and typically low count in samples, demanding high-power microscopy for visualization, which often results in low detection efficiency and potential misdiagnosis (6, 7). Despite advancements in molecular techniques such as polymerase chain reaction (PCR) and RNA scope aimed at improving diagnostic accuracy, universal reliability and acceptance have not been achieved (8, 9).

Advancements in information technology have propelled the integration of AI into the tuberculosis diagnostic workflow. AI, a term originally conceptualized during a seminal conference at Dartmouth in 1956 (10), has evolved significantly, particularly with breakthroughs in convolutional neural networks (11). Emerging evidence underscores AI’s potential to revolutionize medical diagnostics, including tuberculosis, by automating and enhancing the accuracy of traditional detection methods (12).

In tuberculosis diagnostics, the morphology of the tubercle bacillus, characterized by a simple rod shape approximately 4 μm in length and 0.5–1.0 μm in diameter, emerges distinctly following acid-fast staining, appearing purple-red against a blue background (13). This distinct morphological feature lends itself well to machine-learning algorithms for automatic detection. This review aims to synthesize the existing literature on machine-learning-based models for tuberculosis bacilli identification, critically evaluating traditional methodologies while highlighting the transformative potential of AI in this field.

## 2 Onset and progression of tuberculosis

*Mycobacterium tuberculosis*, the pathogen responsible for tuberculosis, is a chronic pathogen that predominantly affects the pulmonary system, although it can also spread to other parts of the body. Tuberculosis’s influence extends beyond mere physical health; it has profoundly affected societal structures and cultural narratives. Despite tuberculosis’s preventability and curability, it continues to pose a substantial global health burden, contributing to significant morbidity and mortality worldwide (14). A comprehensive understanding of the origins and progression of tuberculosis is essential for formulating effective diagnostic, therapeutic, and preventive strategies.

Although direct evidence of tuberculosis in ancient populations is often elusive, advancements in paleopathology have significantly contributed to our understanding. For instance, skeletal lesions indicative of tuberculosis have been documented in Egyptian mummies dating back to approximately 3,300 BCE (15). Similar findings have been observed in archeological samples across Europe, Asia, and the Americas, attesting to the historical global prevalence of the disease (16). Traces of tuberculosis have also been detected in Egyptian mummies since 2400 BCE, underscoring the long-standing interaction of this disease with human populations (17). Known historically as “phthisis” due to the characteristic weight loss in sufferers, tuberculosis was a leading cause of mortality in Europe and North America during the 18th and 19th centuries (18).

The evolutionary history of tuberculosis is a complex topic that remains the subject of intense academic debate. Contemporary research suggests that the *Mycobacterium tuberculosis* complex, which includes *Mycobacterium tuberculosis* and related species, diverged from a common ancestor approximately 70 million years ago (19). However, the precise adaptation of *Mycobacterium tuberculosis* to humans continues to be explored. Two dominant theories exist: the recent emergence model, which suggests that *Mycobacterium tuberculosis* is a zoonotic pathogen that has adapted to humans with the advent of agriculture and denser human settlements (20), and the co-evolution model, which posits a prolonged coexistence and adaptation between *Mycobacterium tuberculosis* complex and human hosts over millennia (21). The identification of *Mycobacterium tuberculosis* by Robert Koch in 1,882 marked a significant advancement in tuberculosis research by clarifying the etiology of the disease (22). Recent genetic analyses have identified specific adaptations in ancient *Mycobacterium tuberculosis* strains, supporting the co-evolutionary hypothesis (19).

Transmission of tuberculosis occurs through inhalation of droplet nuclei infected with *Mycobacterium tuberculosis*, which, upon inhalation, reach the alveoli in the lungs and are phagocytosed by alveolar macrophages. *Mycobacterium tuberculosis* has evolved mechanisms that enable its survival and replication within macrophages, thereby evading host immune responses (23). The host immune system response often results in the formation of granulomas, localized clusters of immune cells that contain bacteria. This usually leads to latent tuberculosis infection (LTBI), wherein the bacteria remain dormant in asymptomatic individuals (24).

Nevertheless, approximately 5%–10% of infected individuals may progress to active tuberculosis, which is characterized by bacteria that overcome immune defenses. Activation can occur shortly after the initial infection or many years later and is typically prompted by immunosuppression or other health challenges (25). Active tuberculosis primarily affects the lungs and causes symptoms such as chronic cough, hemoptysis, chest pain, fever, night sweats, and weight loss. Extrapulmonary tuberculosis, responsible for approximately 15%–20% of tuberculosis cases, can affect other bodily systems, including the lymph nodes, bones, kidneys, and central nervous system (26).

A prominent obstacle in tuberculosis management today is the emergence of drug-resistant *Mycobacterium tuberculosis* strains, primarily due to improper and excessive use of antibiotics, which facilitates the selection of clones with mutations conferring resistance to existing treatments (27). Understanding the evolutionary history of drug resistance is vital for devising effective strategies for drug control. Some evidence suggests that these resistance-conferring mutations could predate the antibiotic era, emphasizing the need to study *Mycobacterium tuberculosi”s* intrinsic evolutionary capabilities (28).

Management of tuberculosis has become increasingly challenging in the face of multi-drug resistant tuberculosis (MDR-TB), which is characterized by resistance to isoniazid and rifampicin, the two primary first-line treatments. Extensively drug-resistant tuberculosis further complicates treatment, involving additional resistance to fluoroquinolones and at least one second-line injectable drug (24). The emergence of drug-resistant tuberculosis is largely attributed to incomplete or inadequate treatment regimens that enable bacteria to survive and develop drug resistance. Factors contributing to this challenge include poor treatment adherence, insufficient access to quality healthcare, and ineffective medication management. As a result, treating drug-resistant tuberculosis necessitates prolonged and costly therapeutic regimens with more significant side effects, posing a considerable challenge to health systems and patients alike (29).

## 3 Global TB management

Global management of tuberculosis is fundamentally based on a tripod that combines effective prevention, prompt diagnosis, and efficient treatment strategies. The World Health Organization underscores the importance of early detection, timely intervention, and preventive approaches as primary components of tuberculosis control programs (30). However, the wide disparity in resource allocation and healthcare infrastructure across various regions poses significant challenges that can lead to inconsistent outcomes in tuberculosis control efforts (31).

In recent years, the application of AI in healthcare has emerged as a transformative strategy that promises to standardize clinical practices and improve health outcomes globally (32). Specifically, in the context of tuberculosis diagnosis, AI technologies offer substantial potential to overcome the limitations of traditional diagnostic methods. Of interest, recent research highlights AI’s capacity to analyze and interpret complex datasets, facilitating accurate and rapid diagnosis of tuberculosis even in resource-limited settings (33).

Artificial intelligence-driven diagnostic tools, particularly those utilizing machine learning and deep learning algorithms, have shown promising results for the identification of tuberculosis from radiographic images. Convolutional neural networks, a class of deep learning algorithms, have achieved remarkable accuracy in detecting pulmonary tuberculosis from chest X-rays, thus providing a non-invasive and cost-effective diagnostic tool (34). Furthermore, AI technologies extend beyond imaging and include natural language processing to analyze clinical notes and other patient data, which may further enhance diagnostic precision (35).

The advent of AI in tuberculosis diagnostics represents not only an improvement in accuracy but also a critical shift toward more accessible diagnostics. Mobile health (mHealth) platforms incorporating AI have the potential to deliver point-of-care diagnostics, particularly in remote and underserved areas, thereby addressing disparities in healthcare access (WHO, 2021). Additionally, AI tools can assist in predicting treatment outcomes and monitoring patient adherence to therapy, thereby optimizing treatment regimens and improving patient management (36).

Despite these advancements, the implementation of AI in tuberculosis diagnosis remains challenging. Ensuring the ethical use of AI and maintaining patient privacy are paramount concerns that require stringent regulatory supervision. Moreover, the deployment of I technologies in low-resource settings requires capacity building and infrastructure development to support and sustain these innovations (37).

Future prospects for AI-driven tuberculosis diagnosis include the development of more robust and generalized algorithms that can be applied across diverse populations and clinical settings. Collaborative efforts among governments, healthcare organizations, and technology firms are essential to harness the full potential of AI in tuberculosis management, ensuring that these advancements translate into tangible health benefits (38).

### 3.1 TB Vaccination and prevention

The Bacille Calmette-Guérin (BCG) vaccine, introduced in the early 20th century, is currently the only licensed tuberculosis vaccine. Despite its widespread use, the BCG vaccine offers limited protection, particularly against tuberculosis in adults. Studies indicate that while BCG effectively reduces the risk of severe tuberculosis in children, its efficacy in adults is minimal (39). Consequently, there is an urgent need for effective vaccines to address the tuberculosis pandemic.

Recent years have witnessed the development of several promising tuberculosis vaccine candidates that are currently undergoing various clinical trials to enhance the efficacy of tuberculosis vaccination.

#### 3.1.1 M72/AS01E vaccine

A recombinant protein vaccine, M72/AS01E, showed encouraging outcomes in a phase IIb trial. The trial demonstrated that the vaccine provides significant protection against pulmonary tuberculosis in adults with latent tuberculosis infection (40).

#### 3.1.2 VPM1002

VPM1002 is a genetically modified BCG vaccine designed to improve the safety and immunogenicity relative to standard BCG vaccines. Ongoing clinical trials aim to establish its effectiveness in preventing tuberculosis (41, 42).

#### 3.1.3 ID93/GLA-SE vaccine

As a recombinant protein vaccine, ID93/GLA-SE demonstrated immunogenicity and protective efficacy in preclinical models. It is currently being evaluated in phase II clinical trials to determine its safety and efficacy in human populations (41). Despite the advances in tuberculosis vaccine development, significant challenges persist. Accurate diagnosis of tuberculosis remains elusive, particularly in children and in cases of extrapulmonary tuberculosis (43). Moreover, the rise of drug-resistant tuberculosis strains poses a serious threat, necessitating innovative therapeutic strategies.

The exploration of host-directed therapies (HDTs) represents an emerging avenue for enhancing tuberculosis treatment. These therapies focus on modulating the host immune response and involve the use of repurposed drugs and immunomodulatory agents. HDTs hold promise for augmenting the efficacy of existing anti- tuberculosis drugs and mitigating disease severity (44).

Substantial progress has been made in the development of vaccines and treatment strategies for tuberculosis. However, ongoing research and global collaboration remain imperative to overcome the challenges posed by tuberculosis and achieve long-term control and eradication of the disease.

### 3.2 Approaches for TB diagnosis

Tuberculosis remains a significant global public health challenge, despite the availability of effective treatments. Prompt and accurate diagnosis is essential to curb its spread and mitigate its impact. Traditional diagnostic methodologies, while foundational, present limitations that modern innovations aim to address. AI has emerged as a transformative technology to enhance tuberculosis detection and management processes, addressing the key limitations inherent in classical diagnostic approaches, as shown in Table 1.

**TABLE 1:** Tuberculosis diagnostic techniques.

Therapies	Technical description		Advantages	Disadvantages	References
Molecular	NAATs	NAATs amplify specific DNA or RNA sequences of *Mycobacterium tuberculosis* (MTB), enabling Rapid detection.	They offer high sensitivity and specificity, particularly in paucibacillary specimens.	However, their reliance on sophisticated Laboratory infrastructure and skilled personnel limits their widespread Accessibility, especially in resource-constrained settings.	(56, 57)
	LPAs	LPAs are molecular assays that detect specific mutations associated with drug resistance in MTB.	They are relatively simple to perform and provide rapid results, making them valuable for drug susceptibility testing.	However, LPAs have limited ability to detect all known drug resistance mutations, potentially leading to inaccurate results.	(58, 59)
Immunological	TST	The TST measures delayed type hypersensitivity to MTB antigens.	It is a low-cost and widely available test, making it suitable for large-scale screening.	However, the TST’s interpretation can be subjective, and it may yield false positive results in individuals with prior BCG vaccination or latent TB infection.	(60)
	IGRAs	IGRAs measure the cellular immune response to specific MTB antigens.	They are blood based tests that are less affected by prior BCG vaccination compared to the TST.	However, IGRAs may have lower sensitivity in immunocompromised individuals and may not distinguish between active TB disease and latent TB infection.	(61)
Advanced methods	CRISPR	WGS provides comprehensive genetic information about MTB strains, enabling the identification of drug resistance mutations, epidemiological tracking, and the detection of emerging drug resistance.	Offers unparalleled resolution	High cost and complex data analysis limit its routine use in clinical settings.	(62, 63)
	Proteomics	Proteomics is the large-scale study of proteins, particularly their structures and functions. It involves analyzing the proteins expressed by the host response to TB infection.	Proteomic approaches offer high specificity and sensitivity, as they can identify unique protein signatures specific to TB, improving diagnostic accuracy.	Such as mass spectrometry, Proteomic technologies are complex and require expensive equipment and specialized expertise, limiting their accessibility in resource-limited settings.	(64, 65)

#### 3.2.1 Sputum smear microscopy

Sputum smear microscopy has long been a cornerstone of tuberculosis diagnosis due to its simplicity and cost-effectiveness. However, its sensitivity ranges between 20% and 60%, significantly affecting its efficacy in cases of low bacterial load such as HIV-positive patients, children, and extrapulmonary tuberculosis (45). Moreover, the manual nature of this technique introduces variability and potential human error.

#### 3.2.2 Culture techniques

Culture remains the gold standard for tuberculosis diagnosis, because of its high sensitivity and specificity. Nonetheless, it requires extended incubation, ranging from 2 to 8 weeks, delaying therapeutic interventions (46). Required technical expertise also limits accessibility in resource-constrained settings.

#### 3.2.3 Chest radiography

While chest radiography provides critical insights into pulmonary tuberculosis, its specificity is limited by similarities in radiographic presentations with other pulmonary conditions (47). Variability in interpretation necessitates skilled radiologists underscore the need for supplementary diagnostic support.

#### 3.2.4 Xpert MTB/RIF assay

This rapid molecular test effectively identifies *Mycobacterium tuberculosis* and rifampicin resistance (48). Despite its advantages, including suitability for point-of-care testing, the assay’s cost and limitations in detecting broader drug resistance pose challenges.

#### 3.2.5 Line probe assays (LPAs)

Line probe assays employ molecular techniques to detect genetic mutations linked to drug resistance, enhancing personalized treatment regimens (49).

#### 3.2.6 Interferon-gamma release assays (IGRAs)

Interferon-gamma release assays offer a distinct advantage over the tuberculin skin test (TST) by minimizing false positives from prior BCG vaccination, although they perform differently in immunocompromised patients.

#### 3.2.7 Whole genome sequencing (WGS)

Whole genome sequencing provides comprehensive insights into *Mycobacterium tuberculosis* strains, aiding epidemiological tracking and drug resistance detection. However, their complexity and cost limit their widespread clinical applications.

#### 3.2.8 Image analysis

Artificial intelligence algorithms have shown high accuracy in interpreting chest radiographs, surpassing human predictions in some studies. For instance, a convolutional neural network can automate the detection of tuberculosis-related abnormalities and reduce dependency on radiological expertise (50).

#### 3.2.9 Predictive modeling

Artificial intelligence-driven predictive models utilize epidemiological data to forecast tuberculosis outbreaks, assess intervention strategies, and optimize resource allocation in tuberculosis control efforts (51, 52).

#### 3.2.10 Natural language processing (NLP)

Natural language processing applications analyze unstructured health data to identify tuberculosis cases and trends, contributing to early detection and monitoring (53, 54).

#### 3.2.11 Digital health integration

Artificial intelligence powered mHealth applications provide adherence support and risk-based patient management, fostering improved treatment outcomes and reducing transmission (55).

While AI’s integration into tuberculosis management holds promise, several challenges persist. Ensuring data privacy and security, addressing algorithmic biases, and securing financial and infrastructure investments remain critical challenges. Collaborative efforts between governments, researchers, and industry stakeholders are essential to harness AI’s full potential in combating tuberculosis.

The integration of AI into tuberculosis diagnostics represents a significant advancement in the fight against this devastating disease. AI can improve tuberculosis detection and management by addressing the limitations associated with traditional methods and enhancing the precision of novel molecular techniques. The pursuit of rapid, accessible, and cost-effective diagnostics in tandem with AI’s transformative capabilities offers substantial promise for enhancing global tuberculosis control.

#### 3.2.12 Advances in TB treatment

Tuberculosis treatment has traditionally involved lengthy courses of antibiotics, typically spanning six months, according to guidelines from the World Health Organization (66, 67). Conventional regimens include drugs, such as isoniazid, rifampicin, pyrazinamide, and ethambutol. However, the emergence of multi-drug-resistant tuberculosis has spurred the development of novel pharmaceuticals.

Bedaquiline and delamanid are among the most recently approved drugs for multi-drug-resistant tuberculosis treatment. These medications have shown promise in improving treatment outcomes and reducing mortality rates in patients with resistant tuberculosis strains (60). Furthermore, recent evidence supports the effectiveness of shorter treatment regimens, ranging between 9 and 12 months, compared with conventional 18 to 24 months courses. These shorter regimens provide equally safe and effective outcomes while being more practical and cost-efficient (68).

## 4 Applications of AI in tuberculosis diagnosis: a beacon of hope

The integration of AI and machine learning into tuberculosis diagnostics represents an innovative frontier in medical research, with the potential to revolutionize traditional diagnostic methodologies (69). The evolving landscape of AI and machine learning offers not only increased accuracy and efficiency but also the promise of scalability and cost-effectiveness, particularly in resource-limited settings where tuberculosis prevalence is high (67).

### 4.1 Chest X-rays (CXR)

Chest radiography remains a cornerstone in the detection of pulmonary tuberculosis. However, its effectiveness is often constrained by the subjective interpretation of radiologists, leading to variability in the diagnosis (70). AI driven solutions, including convolutional neural networks, have consistently demonstrated superior diagnostic performance. For instance, Lakhani and Sundaram (33) leveraged CNNs to classify CXR images with an area under the receiver operating characteristic curve of 0.99, which is a metric they indicate near-perfect accuracy. This suggests a profound potential for AI to augment radiological assessments and improve diagnostic outcomes in tuberculosis screening (35).

### 4.2 Computer-aided detection (CAD)

Artificial intelligence-powered CAD systems have shown promising results for supporting radiologists by pinpointing suspicious lesions indicative of tuberculosis in medical images. Qin et al. (71) illustrated that deep-learning CAD systems surpass traditional methodologies and peer radiologists in diagnostic precision, underscoring AI’s role in efficiently triaging high-risk cases. These systems are invaluable in settings with a dearth of radiological expertise, streamlining workflows, and optimizing resource use (72).

### 4.3 Sputum smear microscopy images

The conventional approach to tuberculosis diagnosis using sputum smear microscopy is labor-intensive and prone to human error (73). By employing machine learning algorithms to examine digital images of ZN-stained smears, enhanced sensitivity and specificity for tuberculosis detection were achieved. This automation reduces human observer bias and enhances diagnostic reliability (74).

### 4.4 Genomic data analysis

Whole genome sequencing (WGS) provides unparalleled insights into *Mycobacterium tuberculosis* at the molecular level. However, the vast amount of generated data, poses analytical challenges that AI can effectively address. Nguyen et al. developed a machine learning model capable of accurately predicting drug resistance, offering a powerful tool for guiding personalized treatment strategies and understanding the epidemiology of drug-resistant tuberculosis (75).

### 4.5 Electronic health records (EHRs)

The wealth of data contained within EHRs can be harnessed by AI and machine learning to uncover predictive patterns and risk factors associated with tuberculosis (76). The application of natural language processing techniques enables the extraction of actionable insights from unstructured clinical notes, potentially integrating tuberculosis diagnostics within routine clinical workflows (77).

### 4.6 Predictive modeling

Artificial intelligence-driven predictive models are instrumental in identifying populations at heightened risk for tuberculosis, thus informing targeted screening and prevention strategies (78, 79). Reddy et al. (80) illustrated the effectiveness of such models in anticipating latent tuberculosis infection risk, which is indispensable for prioritizing at-risk groups in resource-limited settings (81).

### 4.7 Automated microscopy

Artificial intelligence-enhanced automated microscopy systems now rival human microscopists for the accurate detection of bacilli in sputum samples. The system developed by Melendez et al. (82), utilizing deep learning, streamlined the diagnostic process, indicating significant reductions in workload and delays in tuberculosis-endemic regions (83).

### 4.8 Biosensors and wearable devices

Emerging technologies such as AI integrated biosensors offer innovative avenues for tuberculosis detection and monitoring. These devices, capable of analyzing biomarkers in real-time, provide instant diagnostic feedback and are vital for remote areas without an established healthcare infrastructure. Lim et al. (84) introduced a cutting-edge biosensor for detecting *Mycobacterium tuberculosis* DNA, showing potential for on-site diagnostics and continuous patient monitoring (85).

Artificial intelligence’s application in tuberculosis diagnostics is not merely a technological breakthrough but a paradigm shift toward more efficient, accurate, and accessible healthcare solutions. AI stands poised to overcome significant barriers and offers hope to control and eventually overcome tuberculosis. Ongoing efforts to integrate AI into domain must continue to focus on creating adaptable, equitable solutions that prioritize areas with the most pressing needs.

The application of machine learning (ML) methods in the differential diagnosis of latent tuberculosis infection (LTBI) and active tuberculosis (ATB) has been extensively explored in recent studies using transcriptomics and proteomics technologies (cfr. Table 2). Various classification approaches, including decision trees, random forests, support vector machines (SVMs), and Bayesian models, have demonstrated high performance across different cohorts. For instance, a study utilizing decision trees and random forests on transcriptomics data reported an accuracy of 97.8% with a sensitivity of 97.9% in a cohort of TB, LTBI, and healthy controls (HCs). Similarly, unsupervised cluster analysis combined with transcriptomics features achieved an accuracy of 87.8% and specificity of 94.9% in a validation cohort. Furthermore, proteomics-based ML models, such as logistic regression and support vector machines, have also shown promising results. A study employing random forests in proteomics analysis attained a sensitivity of 93.3% in the training cohort and 95% in the validation cohort, with specificity ranging from 80% to 97.7%. Another proteomics-based study integrating neopterin and serum amyloid A with ML techniques yielded a sensitivity of 93.5% and specificity of 94.9% in the validation cohort. These findings underscore the potential of ML-driven approaches in enhancing the accuracy of tuberculosis diagnostics, leveraging multi-omics data to improve clinical decision-making.

**TABLE 2:** List of studies on machine learning (ML) methods and performance obtained based on transcriptomics proteomics technology in the differential diagnosis of latent tuberculosis infection (LTBI) and active tuberculosis (ATB).

Methods	Dataset	Method				
		Accuracy	Recall (sensitivity)	Specificity	References	
Random forest, decision tree.	ATB patients (*n* = 120), LTBI (*n* = 60), HCs (*n* = 20); external cohorts, from the Gambia (*n* = 75), from the Uganda (*n* = 62).	Acc = 82% for Uganda Dataset, Acc = 89% for Gambia dataset	Using a cut-off of 0.8, Uganda: 73%, Gambia: 85%; using a cut-off of 0.6, Uganda: 87%, Gambia: 88%.	Using a cut-off of 0.8, Uganda: 78%, Gambia: 76%; using a cut-off of 0.6, Uganda: 75%, Gambia: 68%.	(86)	ML methods based on transcriptomics technology
Decision tree, random forest, SVM, Bayesian	TB (*n* = 15), LTBI (*n* = 17), HCs (*n* = 15)	97.8%	97.9%	NA	(87)	ML methods based on transcriptomics technology
Decision trees and unsupervised cluster analysis	Identification cohort, ATB (*n* = 28), LTBI (*n* = 25), HCs (*n* = 31); validation cohort, ATB (*n* = 51), LTBI (*n* = 44), HCs (*n* = 35)	87.8%	86.2% with a combination of TNFRSF10C, EBF3, and A2ML	94.9%	(88)	ML methods based on transcriptomics technology
Cluster analysis	Discovery cohort: ATB (*n* = 52), LTBI (*n* = 37), HCs (*n* = 27); validation cohort: ATB (*n* = 205), LTBI (*n* = 123), HCs (*n* = 112)	–	67.3%	91.2%	(89)	ML methods based on transcriptomics technology
Random forest and logistic regression	Discovery cohort: TB (*n* = 146), LTBI (*n* = 146) other diseases (OD) (*n* = 146); validation cohort: TB (*n* = 122), OD (*n* = 127)	–	92% in the test set	71% in the test set	(90)	ML methods based on proteomics technology
Random forest	Discovery cohort: ATB (*n* = 60), LTBI (*n* = 60), HCs (*n* = 60); validation cohort: ATB (*n* = 100), LTBI (*n* = 100), HCs (*n* = 100)	–	93.3% in training cohort and 95% in validation cohort	97.7% in training cohort and 80% in validation cohort	(91)	ML methods based on proteomics technology
Logistic regression	Discovery cohort: ATB *(n* = 20), LTBI (*n* = 40), HCs (*n* = 20); validation cohort: ATB (*n* = 12 + 31), LTBI (*n* = 20 + 20)	–	95% in discovery cohort, 75% and 100% in validation cohort 1 and 2	90% in discovery cohort, 100% and 30% in validation cohort 1 and 2	(92)	ML methods based on proteomics technology
Protein, Neopterin, and serum amyloid A Support vector machine and tree classification	Training cohort: ATB (*n* = 102), HCs (*n* = 91); validation cohort: ATB (*n* = 77), HCs (*n* = 79)	–	93.5%	94.9%	(93)	ML methods based on proteomics technology

## 5 Building an AI-powered TB diagnosis pipeline: from data to deployment

The development of an AI based diagnostic pipeline involves integrating diverse datasets, curating high-quality training images, and employing advanced algorithms for interpreting complex biological data. Interdisciplinary collaboration is pivotal for navigating the challenges of model validation and clinical deployment.

The integration of AI, particularly machine learning, into medical diagnostics has revolutionized the field of infectious diseases, with tuberculosis being the prime focus owing to its significant global burden. A successful machine learning project hinges upon the acquisition and preprocessing of high-quality data. For tuberculosis diagnosis, data can be derived from multiple sources such as chest radiographs, computed tomography scans, sputum smear microscopy images, and clinical data including patient history and laboratory results. Each data type offers unique information that, when integrated, enhances the predictive capability of the machine learning models.

Recent studies have underscored the importance of ensuring data quality through standardization, anonymization, and rigorous preprocessing to maintain patient confidentiality and improve analytical accuracy. Numerous methodologies have been proposed to address issues such as missing values and inconsistencies (94). For example, standardization is required to mitigate the variations stemming from different imaging modalities and settings. Techniques such as histogram equalization and spatial normalization are routinely applied to enhance image quality (95, 96).

Furthermore, data augmentation has been shown to be invaluable, particularly in imaging analyses, because it artificially expands the data set to improve the model generalizability. Techniques such as image rotation, scaling, and horizontal flipping have been extensively utilized in the refinement of convolutional neural networks for tuberculosis detection (8, 9, 97).

**FIGURE 1:**
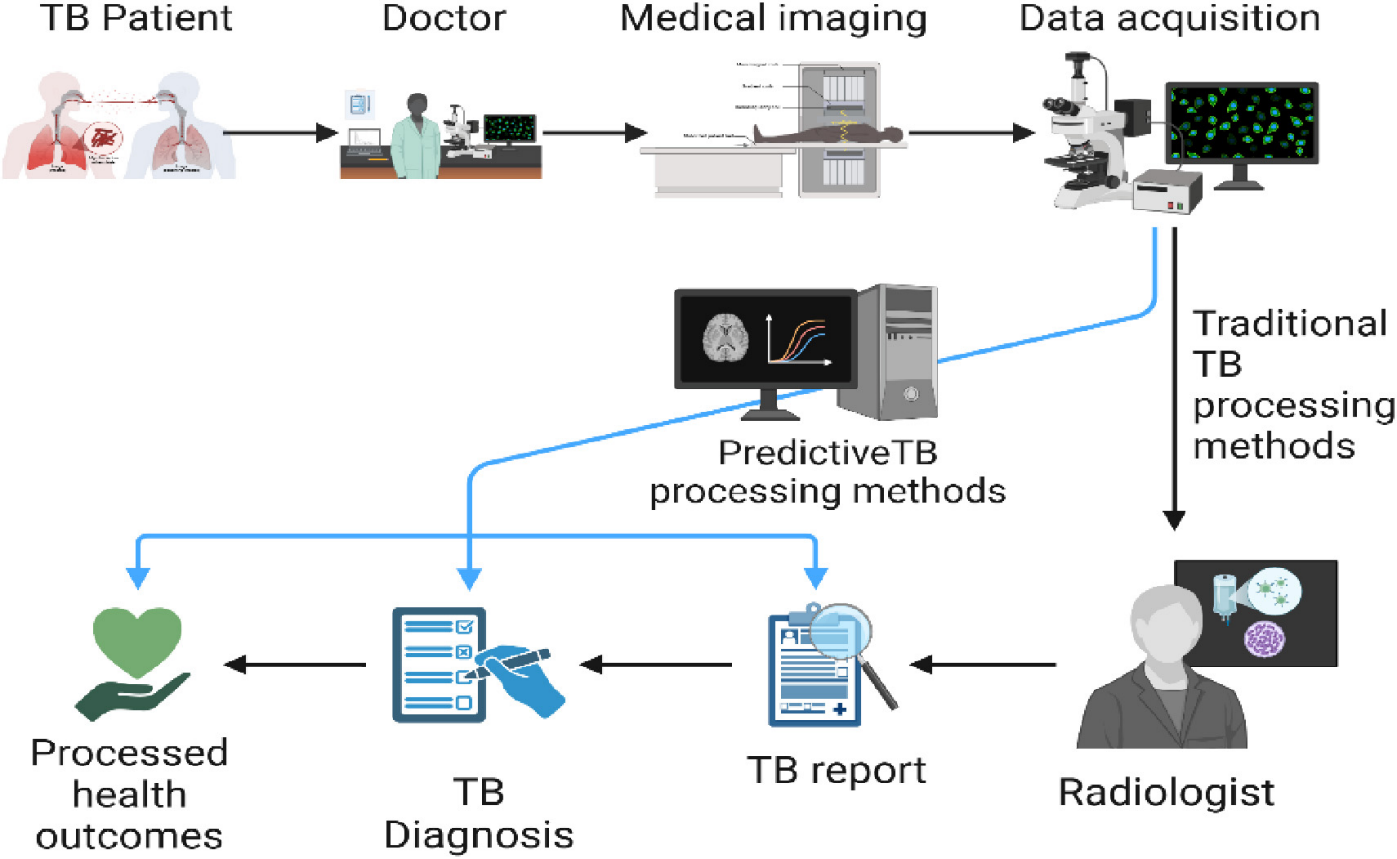
Machine learning pipeline for efficient tuberculosis management and detection.

A robust ML pipeline for tuberculosis diagnosis involves several critical stages, as depicted in Figure 1, starting with data collection and ending with the deployment of the machine learning model in clinical environments. Diverse data sources are paramount, with studies highlighting how variations in data such as ethnic or regional differences can influence the model performance and adaptability (99).

Feature engineering and model selection are pivotal components of the development process. For imaging data, convolutional neural networks have proven particularly efficacious, particularly in extracting relevant features such as lung opacity patterns and nodule characteristics. These machine learning models are adept at discerning complex patterns and minute anomalies indicative of tuberculosis (100). In molecular data analysis, such as nucleic acid amplification tests (NAATs) and whole-genome sequencing, machine learning algorithms have been employed to identify genetic variants linked to tuberculosis strains, aiding precision medicine approaches (101, 102).

The selection of appropriate machine learning models is determined by the nature of the data and specific diagnostic tasks. Although CNNs dominate image processing, algorithms such as support vector machines and random forests are often leveraged for clinical data analysis because of their capability to handle diverse and heterogeneous data types effectively (103, 104). Recent advancements in ensemble learning and neural networks have further expanded the toolbox available for tuberculosis diagnostics (105).

The deployment of machine learning models in clinical settings marks the culmination of developmental processes. Successful integration requires collaboration between AI researchers and healthcare professionals to ensure that model outputs are interpretable and actionable. Importantly, continuous monitoring and updating of machine learning models post-deployment are crucial for maintaining accuracy and adapting to evolving epidemiological patterns (106, 107).

## 6 Conclusion

Despite significant advances in the diagnosis treatment, and prevention approaches, tuberculosis remains a significant global health challenge characterized by considerable morbidity and mortality. The main problem faced by clinicians and tuberculosis programs around the world is the emergence and widespread of drug-resistant tuberculosis, highlighting the urgent need for more effective vaccines, the development of rapid and accurate diagnostic tools, and support for research and innovative projects to identify new efficient therapies. Although traditional diagnostic methods such as sputum smear microscopy and culture remain essential, particularly in resource-limited settings, molecular diagnostics, such as nucleic acid amplification tests and linear probe testing, have become essential for rapid tuberculosis detection and drug resistance testing. Additionally, emerging technologies such as next-generation sequencing and CRISPR-based diagnostics hold promise for the future.

Future directions for AI and machine learning for tuberculosis diagnosis include the development of multimodal AI models that integrate diverse data sources such as imaging, molecular, and clinical data to provide complete diagnostic solutions. Furthermore, AI can play an important role in monitoring and predicting tuberculosis treatment outcomes, thereby enabling personalized treatment plans and improving patient compliance. Robust implementation strategies and continued global collaboration are essential for harnessing the full potential of AI and machine learning for tuberculosis diagnosis.

In conclusion, the integration of AI in the diagnosis and management of tuberculosis offers considerable potential for improving the accuracy, efficiency, and accessibility of diagnosis. However, several challenges remain, including the need for large, high-quality datasets, variability in data quality across different contexts, and integration of these technologies into existing health systems. Addressing these challenges requires collaborative efforts involving researchers, clinicians, and policymakers to standardize data collection and sharing practices and to ensure that AI technologies are accessible and affordable, particularly in resource-limited settings.

## References

[1] Bull F Al-Ansari S Biddle S Borodulin K Buman M Cardon G et al. World Health Organization 2020 guidelines on physical activity and sedentary behaviour. *Br J Sports Med.* (2020) 54:1451–62. 10.1136/bjsports-2020-102955 33239350 PMC7719906

[2] Kyu H Maddison E Henry N Mumford J Barber R Shields C et al. The global burden of tuberculosis: Results from the Global Burden of Disease Study 2015. *Lancet Infect Dis.* (2018) 18:261–84. 10.1111/bjd.15510 29223583 PMC5831985

[3] Yayan J Franke K Berger M Windisch W Rasche K. Early detection of tuberculosis: A systematic review. *Pneumonia.* (2024) 16:11. 10.1186/s41479-024-00133-z 38965640 PMC11225244

[4] Kendall E Shrestha S Dowdy D. The epidemiological importance of subclinical tuberculosis. A critical reappraisal. *Am J Respir Crit Care Med.* (2021) 203:168–74. 10.1164/rccm.202006-2394PP 33197210 PMC7874405

[5] Mathew P. *A Comparative Study on Microscopy, Culture and Xpert MTB/RIF Assay in the Diagnosis of Pulmonary Tuberculosis in Hiv-Infected Patients in a Tertiary Care Hospital.* Bengaluru: Rajiv Gandhi University of Health Sciences (2018).

[6] Musisi E. *Evaluation of the Tuberculosis-Molecular Bacterial Load Assay for Tuberculosis Diagnosis and Monitoring Response to Standard Anti-Tuberculosis Therapy.* Mumbai: University of St Andrews (2023).

[7] Reddy N Kattimani V Swetha G Meiyazhagan G. *Diagnostic Techniques: Clinical Infectious Diseases: Evolving Landscape of Molecular Diagnostics.* Amsterdam: Elsevier (2024). p. 201–25.

[8] Dwivedi S Purohit P Misra R Pareek P Goel A Khattri S et al. Diseases and molecular diagnostics: a step closer to precision medicine. *Indian J Clin Biochem.* (2017) 32:374–98. 10.1007/s12291-017-0688-8 29062170 PMC5634985

[9] Liu Q Jin X Cheng J Zhou H Zhang Y Dai Y. Advances in the application of molecular diagnostic techniques for the detection of infectious disease pathogens (Review). *Mol Med Rep.* (2023) 27:1–14. 10.3892/mmr.2023.12991 37026505 PMC10086565

[10] Rai D. *Artificial Intelligence Through Time: A Comprehensive Historical Review.* Kirtipur: Tribhuvan University (2024).

[11] LeCun Y Bengio Y Hinton G. Deep learning. *Nature.* (2015) 521:436–44. 10.1038/nature14539 26017442

[12] Srivastava V Kumar R Wani M Robinson K Ahmad A. Role of artificial intelligence in early diagnosis and treatment of infectious diseases. *Infect Dis.* (2025) 57:1–26. 10.1080/23744235.2024.2425712 39540872

[13] Ogunbayo A. *Comparison of Methods and Samples Used in the Diagnosis of Childhood PTB and Characterization of Mycobacterium tuberculosis Isolates.* Bloemfontein: University of the Free State (2018). Available online at: https://scholar.ufs.ac.za/items/f09f7e6e-c95f-4773-9050-94f50d620740

[14] Bagcchi S. WHO’s Global Tuberculosis Report 2022. *Lancet Microbe.* (2023) 4:e20. 10.1016/S2666-5247(22)00359-7 36521512

[15] Kerudin A. *Genotyping of Mycobacterium tuberculosis and Mycobacterium leprae ancient DNA.* Oxford: The University of Manchester (2020).

[16] Buzic I Giuffra V. The paleopathological evidence on the origins of human tuberculosis: A review. *J Prev Med Hyg.* (2020) 61(1 Suppl 1):E3–8. 10.15167/2421-4248/jpmh2020.61.1s1.1379 32529097 PMC7263064

[17] Zucconi L. *Ancient Medicine: From Mesopotamia to Rome.* Grand Rapids, MI: Wm. B. Eerdmans Publishing (2019).

[18] Simons J. *Tuberculosis in the Greco-Roman World.* Philadelphia, PA: University of Pennsylvania (2023).

[19] Gagneux S. Ecology and evolution of *Mycobacterium tuberculosis*. *Nat Rev Microbiol.* (2018) 16:202–13. 10.1038/nrmicro.2018.8 29456241

[20] Sobe C. *Zoonotic Transmission Of Tuberculosis Between The Pastoralists And Their Livestock In South Sudan Western Bahr El Ghazal State.* Kampala: Makerere University (2020).

[21] Harkins K. *Pathogen Origins and Evolution in the New World: A Molecular and Bioarchaeological Approach to Tuberculosis and Leishmaniasis.* Tempe, AZ: Arizona State University (2014).

[22] Goetz T. *The remedy: Robert Koch, Arthur Conan Doyle, and the quest to cure tuberculosis.* London: Penguin (2015).

[23] Awuh J Flo T. Molecular basis of mycobacterial survival in macrophages. *Cell Mol Life Sci.* (2017) 74:1625–48. 10.1007/s00018-016-2422-8 27866220 PMC11107535

[24] Hasanuddin A Roswita N Amu I. Immune response toward *Mycobacterium tuberculosis* infection. *Green Med J.* (2020) 2:77–87. 10.33096/gmj.v2i2.47

[25] Venet F Monneret G. Advances in the understanding and treatment of sepsis-induced immunosuppression. *Nat Rev Nephrol.* (2018) 14:121–37. 10.1038/nrneph.2017.165 29225343

[26] Cantres-Fonseca O Rodriguez-Cintrón W Del Olmo-Arroyo F Baez-Corujo S. Extra pulmonary tuberculosis: An overview. *Role Microbes Hum Heal Dis.* (2018) 51:5560. 10.5772/intechopen.81322

[27] Deshpande A Likhar R Khan T Omri A. Decoding drug resistance in *Mycobacterium tuberculosis* complex: Genetic insights and future challenges. *Expert Rev Anti Infect Ther.* (2024) 22:511–27. 10.1080/14787210.2024.2400536 39219506

[28] Gobe I. *Disabling the Intrinsic Resistome of Mycobacterium tuberculosis: Elucidating Hierarchies of DNA Repair and Mutagenesis that Undermine Current Antibiotic Efficacy.* Atlanta, GA: Lyrasis (2021).

[29] Lange C Chesov D Heyckendorf J Leung C Udwadia Z Dheda K. Drug-resistant tuberculosis: An update on disease burden, diagnosis and treatment. *Respirology.* (2018) 23:656–73. 10.1111/resp.13304 29641838

[30] World Health Organization [WHO]. *Global Tuberculosis Report 2024.* Geneva: World Health Organization (2024).

[31] Glaziou P Sismanidis C Floyd K Raviglione M. Global epidemiology of tuberculosis. *Cold Spring Harb Perspect Med.* (2015) 5:a017798. 10.1101/cshperspect.a017798 25359550 PMC4315920

[32] Topol E. High-performance medicine: The convergence of human and artificial intelligence. *Nat Med.* (2019) 25:44–56. 10.1038/s41591-018-0300-7 30617339

[33] Lakhani P Sundaram B. Deep learning at chest radiography: Automated classification of pulmonary tuberculosis by using convolutional neural networks. *Radiology.* (2017) 284:574–82. 10.1148/radiol.2017162326 28436741

[34] Rajpurkar P Irvin J Ball R Zhu K Yang B Mehta H et al. Deep learning for chest radiograph diagnosis: A retrospective comparison of the CheXNeXt algorithm to practicing radiologists. *PLoS Med.* (2018) 15:e1002686. 10.1371/journal.pmed.1002686 30457988 PMC6245676

[35] Esteva A Robicquet A Ramsundar B Kuleshov V DePristo M Chou K et al. A guide to deep learning in healthcare. *Nat Med.* (2019) 25:24–9. 10.1038/s41591-018-0316-z 30617335

[36] Alowais S Alghamdi S Alsuhebany N Alqahtani T Alshaya A Almohareb S et al. Revolutionizing healthcare: The role of artificial intelligence in clinical practice. *BMC Med Educ.* (2023) 23:689. 10.1186/s12909-023-04698-z 37740191 PMC10517477

[37] Shen J Chen J Zheng Z Zheng J Liu Z Song J et al. An innovative artificial intelligence-based app for the diagnosis of gestational diabetes mellitus (GDM-AI): Development Study. *J Med Internet Res.* (2020) 22:e21573. 10.2196/21573 32930674 PMC7525402

[38] Oduoye M Fatima E Muzammil M Dave T Irfan H Fariha F et al. Impacts of the advancement in artificial intelligence on laboratory medicine in low- and middle-income countries: Challenges and recommendations-A literature review. *Health Sci Rep.* (2024) 7:e1794. 10.1002/hsr2.1794 38186931 PMC10766873

[39] Mangtani P Nguipdop-Djomo P Keogh R Trinder L Smith P Fine P et al. Observational study to estimate the changes in the effectiveness of bacillus Calmette-Guérin (BCG) vaccination with time since vaccination for preventing tuberculosis in the UK. *Health Technol Assess.* (2017) 21:1–54. 10.3310/hta21390 28738015 PMC5534974

[40] Tait D Hatherill M Van Der Meeren O Ginsberg A Van Brakel E Salaun B et al. Final analysis of a trial of M72/AS01E vaccine to prevent tuberculosis. *N Engl J Med.* (2019) 381:2429–39. 10.1056/NEJMoa1909953 31661198

[41] Cotton M Madhi S Luabeya A Tameris M Hesseling A Shenje J et al. Safety and immunogenicity of VPM1002 versus BCG in South African newborn babies: A randomised, phase 2 non-inferiority double-blind controlled trial. *Lancet Infect Dis.* (2022) 22:1472–83. 10.1016/S1473-3099(22)00222-5 35772447

[42] Ferluga J Yasmin H Bhakta S Kishore U. Vaccination strategies against *Mycobacterium tuberculosis*: BCG and beyond. *Adv Exp Med Biol.* (2021) 1313:217–40. 10.1007/978-3-030-67452-6_10 34661897

[43] Dheda K Chang K Guglielmetti L Furin J Schaaf H Chesov D et al. Clinical management of adults and children with multidrug-resistant and extensively drug-resistant tuberculosis. *Clin Microbiol Infect.* (2017) 23:131–40. 10.1016/j.cmi.2016.10.008 27756712

[44] Dasanayaka C Dissanayake M. Deep learning methods for screening pulmonary tuberculosis using chest X-rays. *Comput Methods Biomech Biomed Eng Imaging Vis.* (2021) 9:39–49. 10.1080/21681163.2020.1808532

[45] Gopalaswamy R Dusthackeer V Kannayan S Subbian S. Extrapulmonary tuberculosis—an update on the diagnosis, treatment and drug resistance. *J Respir.* (2021) 1:141–64. 10.3390/jor1020015

[46] Naidoo K Perumal R Ngema S Shunmugam L Somboro A. Rapid diagnosis of drug-resistant tuberculosis-opportunities and challenges. *Pathogens.* (2023) 13:27. 10.3390/pathogens13010027 38251335 PMC10819693

[47] Hogan A Jewell B Sherrard-Smith E Vesga J Watson O Whittaker C et al. Potential impact of the COVID-19 pandemic on HIV, tuberculosis, and malaria in low-income and middle-income countries: A modelling study. *Lancet Glob Health.* (2020) 8:e1132–41. 10.1016/S2214-109X(20)30288-6 32673577 PMC7357988

[48] Pai M Behr M. Latent *Mycobacterium tuberculosis* infection and interferon-gamma release assays. *Microbiol Spectr.* (2016) 4. 10.1128/microbiolspec.TBTB2-0023-2016 27763261

[49] Nguyen L Nguyen-Xuan H. Deep learning for computational structural optimization. *ISA Trans.* (2020) 103:177–91. 10.1016/j.isatra.2020.03.033 32303352

[50] Yan C Wang L Lin J Xu J Zhang T Qi J et al. A fully automatic artificial intelligence-based CT image analysis system for accurate detection, diagnosis, and quantitative severity evaluation of pulmonary tuberculosis. *Eur Radiol.* (2022) 32:2188–99. 10.1007/s00330-021-08365-z 34842959 PMC8628489

[51] Margam R. Boosting public health resilience: Harnessing ai-driven predictive analysis to prevent disease outbreaks. *Int J Artif Intell Res Dev.* (2024) 2:76–90. 10.17605/OSF.IO/G4VFB

[52] Nwankwo E Emeihe E Ajegbile M Olaboye J Maha C. Artificial Intelligence in predictive analytics for epidemic outbreaks in rural populations. *Int J Life Sci Res Arch.* (2024) 7:78–94. 10.53771/ijlsra.2024.7.1.0062

[53] Morena D Campos C Castillo M Alonso M Benavent M Izquierdo J. Impact of the COVID-19 pandemic on the epidemiological situation of pulmonary tuberculosis-using natural language processing. *J Pers Med.* (2023) 13:1629. 10.3390/jpm13121629 38138856 PMC10744898

[54] Zhou B Yang G Shi Z Ma S. Natural language processing for smart healthcare. *IEEE Rev Biomed Eng.* (2022) 17:4–18. 10.1109/RBME.2022.3210270 36170385

[55] World Health Organization [WHO]. *Laboratory Testing for 2019 Novel Coronavirus (2019-nCoV) in Suspected Human Cases: Interim Guidance, 17 January 2020.* Geneva: World Health Organization (2020).

[56] Dorman S Schumacher S Alland D Nabeta P Armstrong D King B et al. Xpert MTB/RIF Ultra for detection of *Mycobacterium tuberculosis* and rifampicin resistance: A prospective multicentre diagnostic accuracy study. *Lancet Infect Dis.* (2018) 18:76–84. 10.1016/S1473-3099(17)30691-6 29198911 PMC6168783

[57] Lung T Marks G Nhung N Anh N Hoa N Anh L et al. Household contact investigation for the detection of tuberculosis in Vietnam: Economic evaluation of a cluster-randomised trial. *Lancet Glob Health.* (2019) 7:e376–84. 10.1016/S2214-109X(18)30520-5 30784638

[58] Hillemann D Rüsch-Gerdes S Boehme C Richter E. Rapid molecular detection of extrapulmonary tuberculosis by the automated GeneXpert MTB/RIF system. *J Clin Microbiol.* (2011) 49:1202–5. 10.1128/JCM.02268-10 21270230 PMC3122824

[59] Günther G Saathoff E Rachow A Ekandjo H Diergaardt A Marais N et al. Clinical evaluation of a line-probe assay for tuberculosis detection and drug-resistance prediction in Namibia. *Microbiol Spectr.* (2022) 10:e0025922. 10.1128/spectrum.00259-22 35670620 PMC9241941

[60] Fu A Chan P Cheung Y Moon Y. Dynamic vp-tree indexing for n-nearest neighbor search given pairwise distances. *Vldb J - VLDB.* (2000) 9:154–73. 10.1007/PL00010672

[61] Salgame P Geadas C Collins L Jones-López E Ellner J. Latent tuberculosis infection–Revisiting and revising concepts. *Tuberculosis.* (2015) 95:373–84. 10.1016/j.tube.2015.04.003 26038289

[62] Cohen K Manson A Desjardins C Abeel T Earl A. Deciphering drug resistance in *Mycobacterium tuberculosis* using whole-genome sequencing: Progress, promise, and challenges. *Genome Med.* (2019) 11:45. 10.1186/s13073-019-0660-8 31345251 PMC6657377

[63] Zhang M Lu Y Zhu Y Wu K Chen S Zhou L et al. Whole-genome sequencing to predict *Mycobacterium tuberculosis* drug resistance: A retrospective observational study in Eastern China. *Antibiotics.* (2023) 12:1257. 10.3390/antibiotics12081257 37627677 PMC10451829

[64] Borah K Xu Y McFadden J. Dissecting host-pathogen interactions in TB using systems-based omic approaches. *Front Immunol.* (2021) 12:762315. 10.3389/fimmu.2021.762315 34795672 PMC8593131

[65] Banaei-Esfahani A Nicod C Aebersold R Collins B. Systems proteomics approaches to study bacterial pathogens: Application to *Mycobacterium tuberculosis*. *Curr Opin Microbiol.* (2017) 39:64–72. 10.1016/j.mib.2017.09.013 29032348 PMC5732070

[66] Iqbal S Khan T Naveed K Naqvi S Nawaz S. Recent trends and advances in fundus image analysis: a review. *Comput Biol Med.* (2022) 151(Pt A):106277. 10.1016/j.compbiomed.2022.106277 36370579

[67] World Health Organization [WHO] *Global Tuberculosis Report 2021: Supplementary Material.* Geneva: World Health Organization (2022).

[68] Lönnroth K Mor Z Erkens C Bruchfeld J Nathavitharana R van der Werf M et al. Tuberculosis in migrants in low-incidence countries: Epidemiology and intervention entry points. *Int J Tuberc Lung Dis.* (2017) 21:624–37. 10.5588/ijtld.16.0845 28482956

[69] Gomes S Lopes J Nogueira S. Willingness to pay more for green products: A critical challenge for Gen Z. *J Clean Prod.* (2023) 390:136092. 10.1177/11786361231152438 36741475 PMC9893349

[70] Yadav P. Challenges & solutions for recent advancements in multi-drugs resistance tuberculosis: A review. *Microbiol Insights.* (2023) 16:11786361231152438. 10.1038/s41598-019-51503-3 36741475 PMC9893349

[71] Qin Z Sander M Rai B Titahong C Sudrungrot S Laah S et al. Using artificial intelligence to read chest radiographs for tuberculosis detection: A multi-site evaluation of the diagnostic accuracy of three deep learning systems. *Sci Rep.* (2019) 9:15000. 10.1038/s41598-019-51503-3 31628424 PMC6802077

[72] Annarumma M Withey S Bakewell R Pesce E Goh V Montana G. Automated triaging of adult chest radiographs with deep artificial neural networks. *Radiology.* (2019) 291:196–202. 10.1148/radiol.2018180921 30667333 PMC6438359

[73] Chen W Chang C Lin Y. Pulmonary tuberculosis diagnosis using an intelligent microscopy scanner and image recognition model for improved acid-fast bacilli detection in smears. *Microorganisms.* (2024) 12:1734. 10.3390/microorganisms12081734 39203575 PMC11356913

[74] Tamura G Llano G Aristizábal A Valencia J Sua L Fernandez L. Machine-learning methods for detecting tuberculosis in Ziehl-Neelsen stained slides: A systematic literature review. *Intell Syst Appl* (2024) 22:200365. 10.1016/j.iswa.2024.200365

[75] Nguyen T Huynh T Ren Z Nguyen P Liew A Yin H et al. A survey of machine unlearning. *arXiv Preprint.* (2022). 10.1038/s41746-018-0029-1 31304302 PMC6550175

[76] Rajkomar A Oren E Chen K Dai A Hajaj N Hardt M et al. Scalable and accurate deep learning with electronic health records. *NPJ Digit Med.* (2018) 1:1–10. 10.1038/s41746-018-0029-1 31304302 PMC6550175

[77] Wang Y Wang L Rastegar-Mojarad M Moon S Shen F Afzal N et al. Clinical information extraction applications: A literature review. *J Biomed Inform.* (2018) 77:34–49. 10.1016/j.jbi.2017.11.011 29162496 PMC5771858

[78] Hargreaves J Boccia D Evans C Adato M Petticrew M Porter J. The social determinants of tuberculosis: From evidence to action. *Am J Public Health.* (2011) 101:654–62. 10.2105/AJPH.2010.199505 21330583 PMC3052350

[79] Rasanathan K Sivasankara Kurup A Jaramillo E Lönnroth K. The social determinants of health: Key to global tuberculosis control. *Int J Tuberc Lung Dis.* (2011) 15(Suppl 2):30–6. 10.5588/ijtld.10.0691 21740657

[80] Reddy S. Explainability and artificial intelligence in medicine. *Lancet Digit Health.* (2022) 4:e214–5. 10.1016/S2589-7500(22)00029-2 35337639

[81] Houben R Dodd P. The global burden of latent tuberculosis infection: A Re-estimation using mathematical modelling. *PLoS Med*. (2016) 13:e1002152. 10.1371/journal.pmed.1002152 27780211 PMC5079585

[82] Melendez J Sánchez C Philipsen R Maduskar P Dawson R Theron G et al. An automated tuberculosis screening strategy combining X-ray-based computer-aided detection and clinical information. *Sci Rep*. (2016) 6:25265. 10.1038/srep25265 27126741 PMC4850474

[83] Reeve B McFall S Song R Warren R Steingart K Theron G. Commercial products to preserve specimens for tuberculosis diagnosis: A systematic review. *Int J Tuberc Lung Dis*. 2018;22:741–53. 10.5588/ijtld.17.0816 29914599

[84] Joshi H Kandari D Maitra S Bhatnagar R. Biosensors for the detection of *Mycobacterium tuberculosis*: A comprehensive overview. *Crit Rev Microbiol*. (2022) 48:784–812. 10.1080/1040841X.2022.2035314 35196464

[85] dos Santos C Lucena G Pinto G Júnior M Marques R. Advances and current challenges in non-invasive wearable sensors and wearable biosensors—A mini-review. *Med Devices Sensors*. (2021) 4:e10130. 10.1002/mds3.10130

[86] Maertzdorf J McEwen G Weiner J Tian S Lader E Schriek U et al. Concise gene signature for point-of-care classification of tuberculosis. *EMBO Mol Med*. (2016) 8:86–95. 10.15252/emmm.201505790 26682570 PMC4734838

[87] Lee S Wu L Huang G Huang K Lee T Weng J. Gene expression profiling identifies candidate biomarkers for active and latent tuberculosis. *BMC Bioinformatics.* (2016) 17 (Suppl 1):3. 10.1186/s12859-015-0848-x 26818387 PMC4895247

[88] Wang S He L Wu J Zhou Z Gao Y Chen J et al. Transcriptional profiling of human peripheral blood mononuclear cells identifies diagnostic biomarkers that distinguish active and latent tuberculosis. *Front Immunol*. (2019) 10:2948. 10.3389/fimmu.2019.02948 31921195 PMC6930242

[89] Li Z Hu J Liu P Cui D Di H Wu S. Microarray-based selection of a serum biomarker panel that can discriminate between latent and active pulmonary TB. *Sci Rep*. (2021) 11:14516. 10.1038/s41598-021-93893-3 34267288 PMC8282789

[90] Morris T Hoggart C Chegou N Kidd M Oni T Goliath R et al. Evaluation of host serum protein biomarkers of tuberculosis in sub-Saharan Africa. *Front Immunol*. (2021) 12:639174. 10.3389/fimmu.2021.639174 33717190 PMC7947659

[91] Li J Wang Y Yan L Zhang C He Y Zou J et al. Novel serological biomarker panel using protein microarray can distinguish active TB from latent TB infection. *Microbes Infect*. (2022) 24:105002. 10.1016/j.micinf.2022.105002 35598729

[92] Delemarre E van Hoorn L Bossink A Drylewicz J Joosten S Ottenhoff T et al. Serum biomarker profile including CCL1, CXCL10, VEGF, and adenosine deaminase activity distinguishes active from remotely acquired latent tuberculosis. *Front Immunol*. (2021) 12:725447. 10.3389/fimmu.2021.725447 34691031 PMC8529994

[93] Agranoff D Fernandez-Reyes D Papadopoulos M Rojas S Herbster M Loosemore A et al. Identification of diagnostic markers for tuberculosis by proteomic fingerprinting of serum. *Lancet*. (2006) 368:1012–21. 10.1016/S0140-6736(06)69342-2 16980117 PMC7159276

[94] Diaz O Kushibar K Osuala R Linardos A Garrucho L Igual L et al. Data preparation for artificial intelligence in medical imaging: A comprehensive guide to open-access platforms and tools. *Phys Med*. (2021) 83:25–37. 10.1016/j.ejmp.2021.02.007 33684723

[95] Taylor R Melnick E Fleishman W Venkatesh A. The impact of risk standardization on variation in CT use and emergency physician profiling. *AJR Am J Roentgenol*. (2018) 211:392–9. 10.2214/AJR.17.19188 29975119

[96] Mali S Ibrahim A Woodruff H Andrearczyk V Müller H Primakov S et al. Making radiomics more reproducible across scanner and imaging protocol variations: A review of harmonization methods. *J Pers Med*. (2021) 11:842. 10.3390/jpm11090842 34575619 PMC8472571

[97] Kumar T Brennan R Mileo A Bendechache M. Image data augmentation approaches: A comprehensive survey and future directions. *IEEE Access.* (2024) 12:187536-187571. 10.1109/ACCESS.2024.3470122

[98] Nessipkhanov D Davletova V Kurmanbekkyzy N Omarov B. Deep CNN for the identification of pneumonia respiratory disease in chest X-Ray imagery. *Int J Adv Comput Sci Appl*. (2023) 14:652–61. 10.29210/1202424847

[99] Mariyono D Abror M. AI in enhancing cultural sensitivity. *J Educ J Pendidik Indones*. (2024) 10:296–310. 10.1109/ACCESS.2019.2920980

[100] Monkam P Qi S Ma H Gao W Yao Y Qian W. Detection and classification of pulmonary nodules using convolutional neural networks: A survey. *IEEE Access*. (2019) 7:78075–91. 10.1109/ACCESS.2019.2920980

[101] Hwang E Jeong W David P Arentz M Ruhwald M Yoon S. AI for Detection of Tuberculosis: Implications for Global Health. *Radiol Artif Intell*. (2024) 6:e230327. 10.1148/ryai.230327 38197795 PMC10982823

[102] Chopra K Singh S. Tuberculosis: Newer diagnostic tests: Applications and limitations. *Indian J Tuberc*. (2020) 67:S86–90. 10.1016/j.ijtb.2020.09.025 33308677

[103] Shailaja K Seetharamulu B Jabbar M. Machine learning in healthcare: A review. In: *Proceedings of the 2018 Second International Conference on Electronics, Communication and Aerospace Technology (ICECA).* Coimbatore: IEEE (2018). p. 910–4.

[104] Ngiam K Khor I. Big data and machine learning algorithms for health-care delivery. *Lancet Oncol*. (2019) 20:e262–73. 10.1016/S1470-2045(19)30149-4 31044724

[105] Hrizi O Gasmi K Ben Ltaifa I Alshammari H Karamti H Krichen M et al. Tuberculosis disease diagnosis based on an optimized machine learning model. *J Healthc Eng*. (2022) 2022:8950243. 10.1155/2022/8950243 35494520 PMC9041161

[106] Aguilar-Gallardo C Bonora-Centelles A. Integrating artificial intelligence for academic advanced therapy medicinal products: Challenges and opportunities. *Appl Sci*. (2024) 14:1303. 10.3390/app14031303

[107] Anner R Agrly Y. Integrating artificial intelligence into clinical practice guidelines: Opportunities and challenges. *J Midwifery Hist Philos*. (2024) 1:7–13.

